# The pangenome of banana highlights differences between genera and genomes

**DOI:** 10.1002/tpg2.20100

**Published:** 2021-07-05

**Authors:** Habib Rijzaani, Philipp E. Bayer, Mathieu Rouard, Jaroslav Doležel, Jacqueline Batley, David Edwards

**Affiliations:** ^1^ School of Biological Sciences and Institute of Agriculture University of Western Australia Perth WA Australia; ^2^ Indonesian Agency for Agricultural Research and Development Jakarta Indonesia; ^3^ Bioversity International Parc Scientifique Agropolis II Montpellier 34397 France; ^4^ Institute of Experimental Botany of the Czech Academy of Sciences Centre of the Region Hana for Biotechnological and Agricultural Research Šlechtitelů 31 Olomouc 77900 Czech Republic

## Abstract

Banana (Musaceae family) has a complex genetic history and includes a genus *Musa* with a variety of cultivated clones with edible fruits, *Ensete* species that are grown for their edible corm, and monospecific *Musella* whose generic status has been questioned. The most commonly exported banana cultivars belong to Cavendish, a subgroup of *Musa* triploid cultivars, which is under threat by fungal pathogens, though there are also related species *M. balbisiana* Colla (B genome), *M. textilis* Née (T genome), and *M. schizocarpa* N. W. Simmonds (S genome), along with hybrids of these genomes, which potentially host genes of agronomic interest. Here we present the first cross‐genus pangenome of banana, which contains representatives of the *Musa* and *Ensete* genera. Clusters based on gene presence–absence variation (PAV) clearly separate *Musa* and *Ensete*, while *Musa* is split further based on species. These results present the first pangenome study across genus boundaries and identifies genes that differentiate between Musaceae species, information that may support breeding programs in these crops.

## Core Ideas

- We assembled the first banana pangenome across two genera.
- The two genera exhibit high levels of divergence.
- The banana pangenome contains very few novel disease resistance genes.

**Abbreviations**
BUSCO: benchmarking universal single‐copy orthologs
GO: gene ontology
NLR: nucleotide binding‐site leucine rich repeat
PAV: presence–absence variation
RGA: resistance gene analog
SNP: single nucleotide polymorphism.

## 1 INTRODUCTION

The banana family (Musaceae) is a monophyletic clade comprising of three genera, *Musa*, *Ensete*, and *Musella* (Kress, 1990). Most of the identified species of this family belong to the *Musa* genus, which includes the edible fruit‐bearing banana cultivars, with the latest database of *Musa* Germplasm Information System recording 6,548 accessions that are maintained in 29 collections around the world (Ruas et al., 2017). In contrast, the *Ensete* genus is comprised of cultivars with nonedible fruits (Oyen & Lemmens, 2002). The corm or the basal pseudo‐stem of the *Ensete* plants is used as a starch‐rich food source, predominantly in Ethiopia, where it plays significant agricultural and economic roles. There are six known species within the *Ensete* genus (Ploetz et al., 2007), with 387 accessions characterized by Yemataw et al. (2017). *Musella* is monotypic occurring only in higher altitudes in southwestern China (Liu et al., 2003) and may be monophyletic with *Ensete* (Liu et al., 2010).

In contrast to the limited geographical distribution of *Ensete*, banana from the genus *Musa* is widely produced across the tropics and subtropics, cultivated on more than 5.6 million ha and produces about 114 Tg of fruit annually (FAO, 2018). The main banana producing countries are from southern, eastern, and southeastern Asia; central and eastern Africa; as well as Central America and the Caribbean. Although there are hundreds of banana cultivars around the globe, few are grown commercially for large‐scale production, with the main commercial banana being triploid clones from the Cavendish subgroup such as ‘Grand Naine’ and ‘Robusta’ (FAO, 2019).

Cultivation of banana is threatened by both biotic and abiotic stress, and most banana cultivars are prone to cold damage, drought, or salinity (van Asten et al., 2011). Pests and pathogens can threaten banana cultivation, including Fusarium wilt, black leaf streak disease, banana bunchy top disease, and Moko disease (Ploetz et al., 2015). These diseases can spread quickly because of the monoculture practice of banana cultivation. This is exemplified by the collapse of the ‘Gros Michel’ based banana trade in the 1960s caused by the spread of *Fusarium oxysporum* f. sp. *cubense* (Robinson, 1996). The Cavendish banana that replaced Gros Michel after the epidemic is now being threatened by the tropical race 4 (TR4) pathotype of the same pathogen (Ploetz et al., 2015).

Banana breeding and cultivar improvement are being hampered by banana's long generation time and low fertility. Despite this, successful hybrids were produced by conventional cross‐breeding at FHIA, EMBRAPA, and some other breeding programs (Escalant et al., 2002) but the level of adoption of the hybrids has remained low (Thiele et al., 2021). Tissue‐culture‐based approaches such as in vitro embryo rescue, genetic engineering, or genome editing techniques could overcome some of these problems (Tripathi et al., 2015), and some reports indicate the successful production of transgenic banana with improved agronomic traits, including resistance to biotic stress (Ghag et al., 2015; Tripathi et al., 2017) and better postharvest handling (Elitzur et al., 2016). While most transgenic banana improvement remains in the laboratory (Dale et al., 2017), some field trials are underway in banana producing countries including Australia, Uganda, and the Philippines (Paul et al., 2018).

As the cost of DNA sequencing continues to decline, increasing quantities of genetic and genomic information are becoming available for banana and its relatives. There are currently seven reference genome assemblies available for the *Musa* genus. The *M. acuminata* ssp. *malaccensis* (Ridl.) N. W. Simmonds genome, derived from a doubled‐haploid Pahang accession, represents the banana A genome (*n* = 11) (D'Hont et al., 2012; Martin et al., 2016), whereas *M. balbisiana* Colla, derived from a Pisang Klutuk Wulung accession, represents the B genome (*x* = 11) (Davey et al., 2013; Wang et al., 2019). There is also an assembled genome for *M. itinerans* Cheesman, a cold‐tolerant banana cultivar from the Yunan region in China (Wu et al., 2016). More recently, three genomes from different subspecies of *M. acuminata*, namely ssp. *banksii* N. W. Simmonds, *zebrina* (Van Houtte ex Planch.) Nasution, and *burmannica* N. W. Simmonds were published (Rouard et al., 2018), while another publication presented a chromosome‐scale assembly of the *M. schizocarpa* genome (S genome, *x* = 11) (Belser et al., 2018). This latest genome assembly benefited from the incorporation of long‐read sequences from third‐generation genome sequencing. These assemblies cover all the known genome types of *Musa* banana, except *Australimusa* (T genome, *x* = 10).

**Core Ideas**

- We assembled the first banana pangenome across two genera.
- The two genera exhibit high levels of divergence.
- The banana pangenome contains very few novel disease resistance genes.

Genomic resources for *Ensete* (*x* = 9) are also becoming available. One draft reference genome assembly has been published for an unknown variety of *E. ventricosum* (Welw.) Cheesman, together with three assemblies for known Ethiopian cultivars: Derea, Bedadeti, and Onjamo (Harrison et al., 2014). Whole‐genome sequencing data is also available for a further 17 *E. ventricosum* accessions representing 15 varieties (Yemataw et al., 2018), though no whole‐genome sequence data is available for other species of *Ensete*.

To complement this public information, we have generated Illumina next‐generation sequencing data for nine banana accessions with an average coverage of 55×. These accessions include *M. acuminata* species, some hybrids with B‐genome banana with various ploidy levels, and a variety of *Fe'i* banana (*Australimusa/Callimusa*, T genome), which is thought to be domesticated independently from both *M. acuminata* and its hybrids with *M. balbisiana* and is characterized by its upright fruit bunch and fruit with high‐carotenoid content (Sharrock et al., 2001). Using this data, we produced a draft cross‐genus pangenome that captures the diversity of banana genome types and highlights the diversity of gene content across the Musaceae, identifying genes that may play a role in the evolutionary differentiation between *Ensete* and *Musa* genera as well as variable genes that could be used to improve the agronomic performance and disease resistance in this important family.

## 2 MATERIALS AND METHODS

Genomic short reads of diverse banana accessions from two genera of Musaceae, *Musa* and *Ensete*, were collected from both publicly available data and newly generated from banana samples from Indonesian Agricultural Agency for Research and Development at the Indonesian Tropical Fruit Research Institute germplasm collection. The metadata of the reads used in the banana pangenome construction and analysis is listed in Supplemental Tables S1 and S2.

We applied the previously published iterative pangenome assembly pipeline (Golicz et al., 2016; Montenegro et al., 2017; Zhao et al., 2018). Bowtie2 v2.3.3.1 (‐I 0 ‐X 1000 –end‐to‐end –sensitive) (Langmead & Salzberg, 2012) and SAMtools v1.2 (Li et al., 2009) were used for read mapping and subsequent extraction of unmapped reads. We assembled the pangenome in 14 iterations, first starting with the five *M. acuminata* individuals with a coverage above 10×, then merged the remaining <10× coverage *M. acuminata* individuals into one assembly in step six, then added the remaining *Musa* and *Ensete* individuals in separate steps. The order of iterations is shown in Supplemental Table S2. The assembly of additional pangenome contigs was conducted using MaSuRCA v3.1.3 (Zimin et al., 2013). Contaminant (nonplant) contigs were discarded by comparing all assembled contigs with NCBI‐NR using BLAST and discarding contigs with the highest‐scoring hits with nonplants.

Repeat elements were identified and masked using RepeatMasker v4.0.6 (Smit et al., 2015) and gene models were generated using MAKER v.2.31.8 (Holt & Yandell, 2011) and AUGUSTUS v3.2.2 (Stanke et al., 2006). Protein, expressed sequence tag, and transcriptome data were collected from the Sequence Read Archive and used as evidence for gene models (Supplemental Table S3). Gene ontology (GO) terms were assigned to the identified gene models using Blast2GO (Conesa et al., 2005). The GO‐term enrichment used Blast2GO and topGO (Alexa & Rahnenführer, 2009). Benchmarking universal single‐copy orthologs (BUSCO) (Simão et al., 2015) from OrthoDB (www.orthodb.org) analysis used 1,440 orthologs from the plant database (embryophyta_odb9) (Zdobnov et al., 2017).

Single nucleotide polymorphisms (SNPs) were called following Bowtie2 read mapping using Bcftools v1.2‐63 (Li, 2011) and were quality processed (SAMtools mpileup ‐q 30 ‐Q 20). Functional annotation of the final SNPs was carried out using the SnpEff tool (Cingolani et al., 2012).

To call presence–absence variations (PAVs), reads from all samples were mapped to the pangenome using Bowtie2, and SGSGeneLoss was used to call PAVs using standard settings (Golicz et al., 2015). Samples with a coverage below 15× were excluded. PVClust (Suzuki & Shimodaira, 2006) was used for hierarchical clustering based on the gene presence–absence matrix. Core and variable gene counts were plotted using PanGP (Y. Zhao et al., 2014). This was also used for the modeling and prediction of the pangenome size. vcftools (Danecek et al., 2011) was used to calculate Weir‐Cockerham's *F*_ST_ values at 100K nucleotide windows across the pangenome, which were visualized using R. Genes were classified into resistance gene analogs (RGAs) by using RGaugury (Li et al., 2016).

The data generated for this project is available in the Sequence Read Archive under BioProject ID PRJNA612747. All assemblies and annotations generated are available at the Banana Genome Hub (Droc et al., 2013) at https://banana-genome-hub.southgreen.fr and https://doi.org/10.26182/y00m-nb33.

## 3 RESULTS AND DISCUSSION

We have assembled the first pangenome of the banana family (Musaceae) using the iterative mapping and assembly approach (Golicz et al., 2016), representing three *Musa* species, a diverse group of cultivated banana clones and one *Ensete* species. This approach requires the selection of an initial reference assembly. There are seven draft or reference genomes published for *Musa*, including reference assemblies of *M. acuminata* spp. *malaccensis* (A genome), *M. schizocarpa* (S genome), and *M. balbisiana* (B genome), a draft assemblies of *M. itinerans*, as well as three draft assemblies from different subspecies of *M. acuminata*, namely ssp. *banksii, zebrina and burmannica* (A genome) (D'Hont et al., 2012; Davey et al., 2013; Wu et al., 2016; Belser et al., 2018; Rouard et al., 2018). In addition, there are four genome assemblies for *Ensete* at the scaffold level (Yemataw et al., 2018) (Supplemental Table S4). Analysis of the assembly statistics suggests that the *M. acuminata* spp. *malaccensis* and *M. schizocarpa* genome assemblies are the most contiguous. The *M. acuminata* assembly showed the most complete BUSCO orthologs for both single‐copy and duplicated genes (Supplemental Table S5; Supplemental Figure S1) and so was selected as the base reference for iterative pangenome assembly. Additional public sequence data is listed in Supplemental Table S1.

Whole‐genome Illumina sequence data was collated from 15 banana accessions, 12 of which represent the genus *Musa* with three from *Ensete*. The *Musa* accessions include six representatives of *M. acuminata* (A genome), one from *M. balbisiana* (B genome), *M. itinerans*, and *M. Fe'I* (T genome) as well as three A‐B genome hybrids (one AB and two AAB). The reference‐guided iterative assembly approach (Golicz et al., 2016) produced 510,493 scaffolds with a total length of 672 Mb in addition to the *M. acuminata* reference genome of 451 Mb (A genome). The assembled pangenome produced a slight increase in BUSCO score compared with the single reference genome (Supplemental Table S6). This is in line with other pangenomes; for example, in the rape (*Brassica napus* L.) pangenome the assembled pangenome contained a single additional complete BUSCO not present in the reference genome (Hurgobin et al., 2018).

Gene prediction identified 12,310 candidate protein‐coding gene models in the newly assembled contigs in addition to 35,276 gene models already identified in the *M. acuminata* genome. Other plant pangenome studies have identified a much smaller proportion of additional sequence. For example, the rice (*Oryza sativa* L.) pangenome, which was constructed from thousands of cultivars, only identified 80 Mb of novel sequence, equal to 30% of the reference genome (Zhao et al., 2018). The hexaploid wheat (*Triticum aestivum* L.) pangenome identified 350 Mb of additional sequence (Montenegro et al., 2017), an increase in size of <10%. Similarly, the kale (*B. oleracea* L.) pangenome size is ∼25% larger than the reference *B. oleracea* genome (Golicz et al., 2016). These previous pangenomes were assembled within a specific genus, or with species of the same genome, and the large pangenome size we observe in banana is most likely a result of the inclusion of multiple *Musa* species as well as the *Ensete* genus. The inclusion of several cultivated *Musa* may also contribute to the expansion of the pangenome size, as their genomes diverged, and include admixture with other genomes during domestication (Martin et al., 2020). This broader approach permits the examination of gene presence or absence across diverse Musaceae and highlights the applicability of cross‐genera pangenomics.

Following construction of the pangenome, we remapped the original next‐generation sequencing reads to the pangenome and identified gene PAV using SGSGeneloss (Golicz et al., 2015). On average, there are 34,014 genes present in each of the Musaceae individuals, with the smallest number observed for sample Ensete01 (27,452) and highest observed for Bile (Bire) with (37,091). All *Ensete* samples appear to have fewer genes than *Musa* samples (Supplemental Table S7). While the number of genes identified for *Musa* species is similar to the number of genes in the *M. acuminata* reference genome, the number of predicted genes in *Ensete* species is less than the 42,749 genes reported for the draft genome of *E. ventricosum*; however, this number could be inflated because of the fragmented nature of the *E. ventricosum* draft genome assembly (Harrison et al., 2014).

**FIGURE 1:**
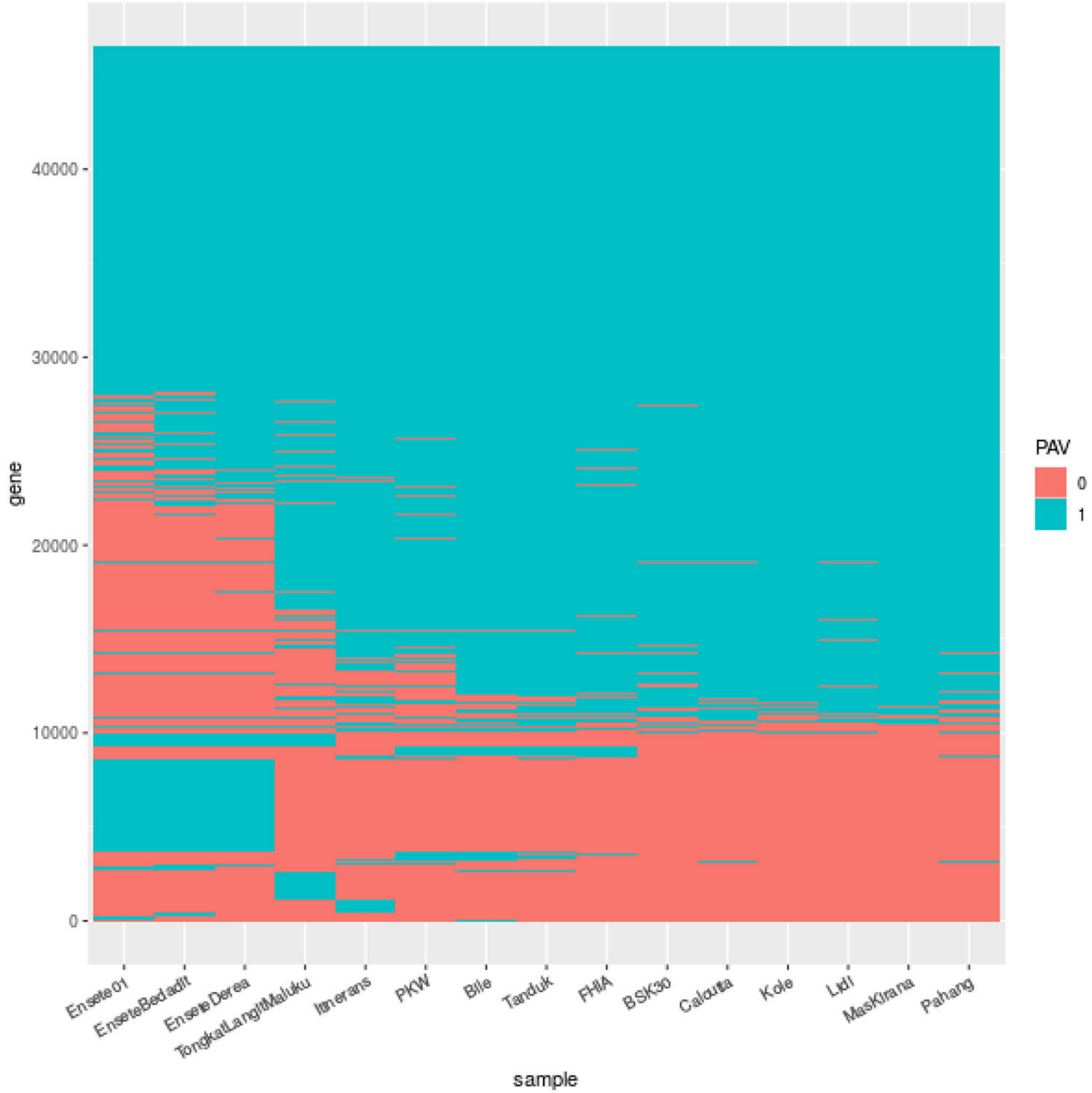
The presence or absence of genes among the banana accessions. Genes are ordered in decreasing order by the number of shared genes among all accessions

**FIGURE 2:**
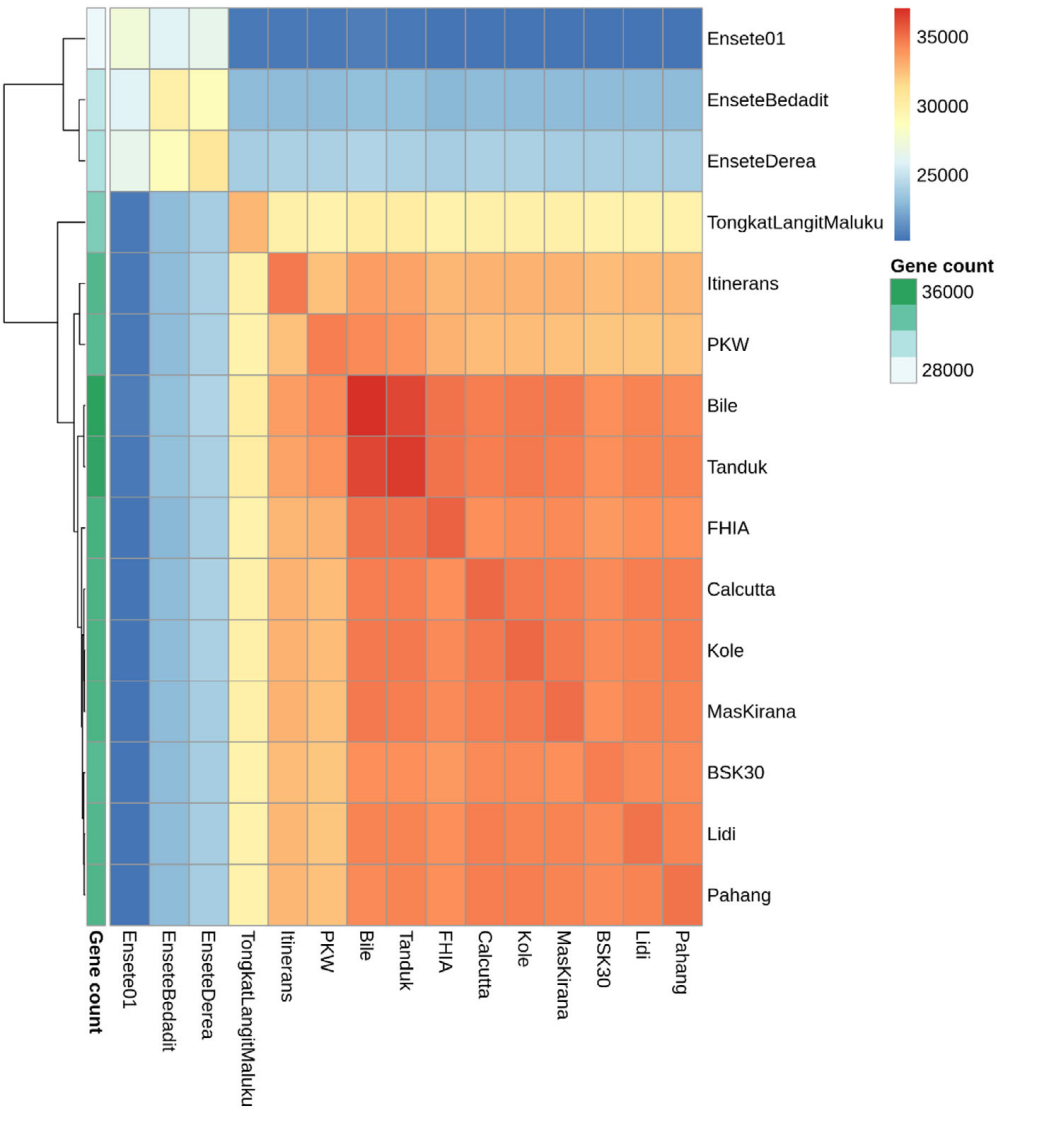
Matrix of numbers of shared genes among the banana accessions used for the pangenome study

Genes showed distinct presence–absence patterns that reflect the divisions of the banana genomes. The set of genes shared by *Musa* species are separate from the set shared by *Ensete* species (Figure 1), and the matrix of shared genes among banana accessions also shows two distinct clusters based on genus (Figure 2). The majority of genes (30,119) are found in both *Ensete* and *Musa*, while 5,629 are present only in the three *Ensete* individuals and 11,924 are present only in *Musa* individuals.

**FIGURE 3:**
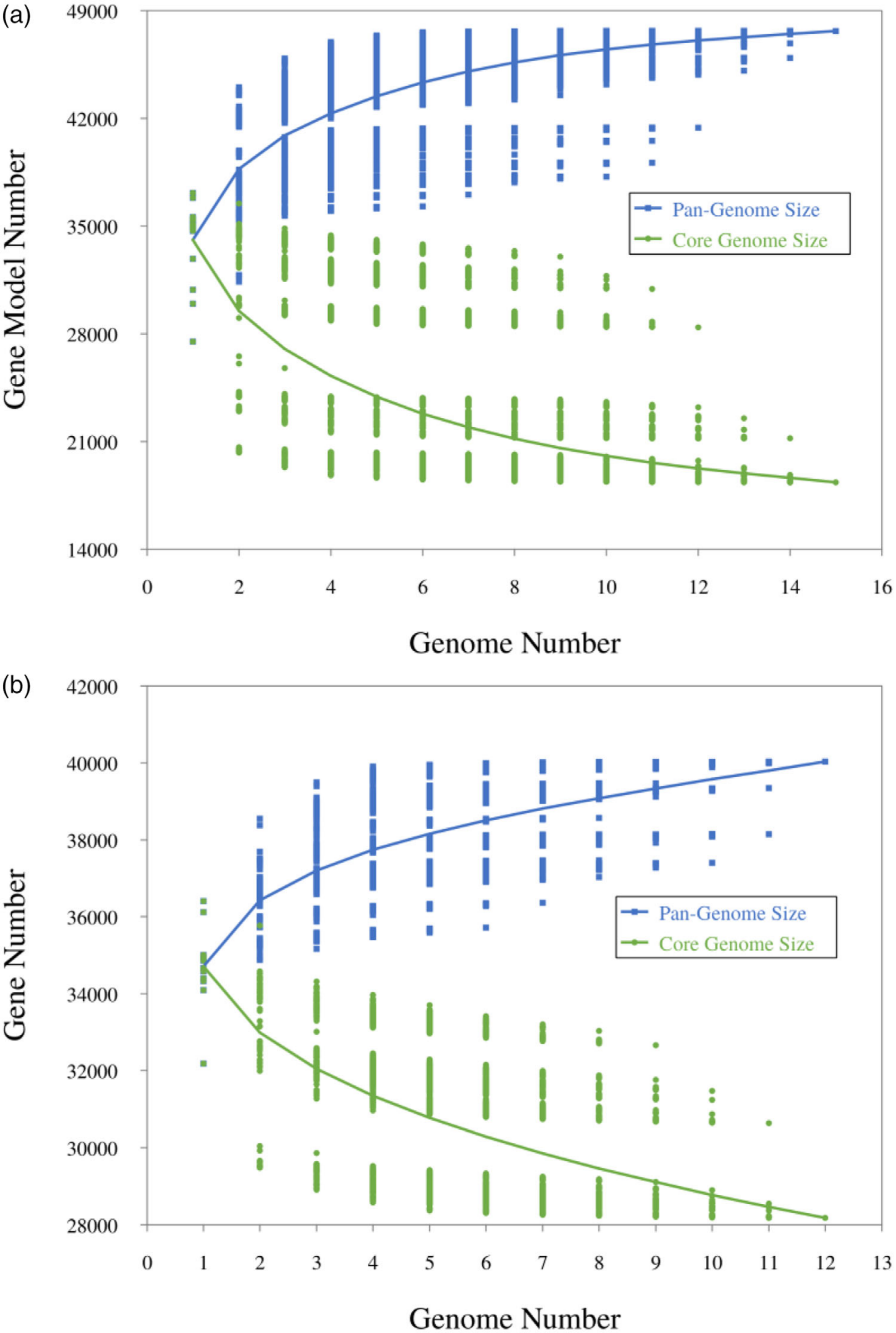
Gene content modeling showing core and pan‐genome size growths. (a) All samples, both from *Musa* and *Ensete* genera, were incorporated into the model. (b) Gene content modeling based on *Musa* accessions only

Gene PAV of the Musaceae pangenome showed a similar trend to previous studies among Musaceae. A study of gene content for the published *E. ventricosum* draft genome by Harrison et al. (2014) found 662 gene models of *M. acuminata* that are absent in *E. ventricosum*, and 9,967 gene models in the *E. ventricosum* genome that were not found in the banana proteome database, though, as the authors acknowledge, the published *E. ventricosum* gene model content may be inflated. Prediction of the banana pangenome size using these gene models and PAV data suggest a core gene number of 18,288 (±29). The observed number of core genes was 18,359 (Figure 3A), suggesting that we have defined the majority of the core gene content. The number of observed variable genes was 29,331 (Figure 3A). Restricting the analysis to within the *Musa* genus (12 samples) we predict a larger number of core genes (27,858 ± 69), with the difference reflecting genes that appear to be conserved in *Musa* but absent from *Ensete* (Figure 3B).

Principal component analysis of the PAV data revealed sample clustering consistent with the genus divisions, with banana samples in the *Musa* genus separate from the *Ensete* genus (Supplemental Figure S2). Subclustering within *Musa* clearly follows subgenome delineation. For example, doubled‐haploid cultivar Pahang (A genome) is separated from cultivar Pisang Klutuk Wulung (diploid, B genome) and cultivar Tongkat Langit Maluku (T genome). The SNP‐based clustering shows highly similar patterns (Supplemental Figure S3). *Musa acuminata* groups are separated into two subclusters based on PAV (Supplemental Figure S4), with one group being A genome individuals and the other group being composed of cultivars Tanduk and Bile (AAB and AB respectively) as well as the improved cultivar FHIA 25 (AAB).

**FIGURE 4:**
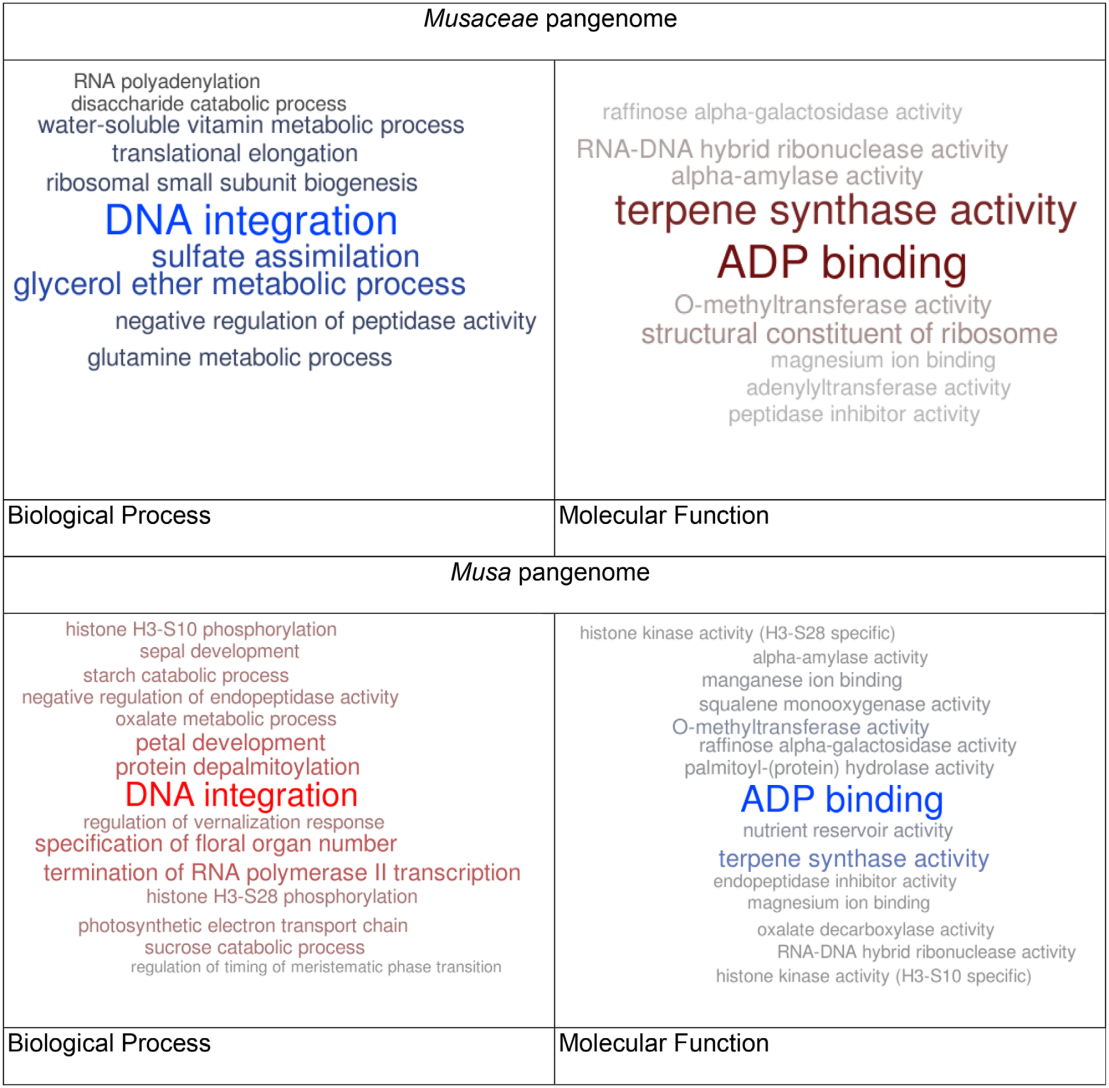
Gene ontology terms overrepresented in the variable regions of the banana pangenome

Gene ontology enrichment analysis was performed to characterize the variable genes within the Musaceae and the *Musa* genus samples (Figure 4; Supplemental Tables S8 & S9). This highlighted diverse terms associated with metabolism across the Musaceae. Interestingly, terms related to flowering, meristem regulation, and nutrient metabolism are identified as enriched in the variable genes among *Musa* genus samples, and these functions may reflect the morphological diversity among *Musa* species as exemplified by various size, shape, color, texture, and taste of the fruit. The differences in enrichment of genes related to flowering could also reflect the difference in flowering between genus *Musa* and *Ensete*, with flowering very delayed in *Ensete* plants (Tsegaye & Struik, 2002).

Genomic variation in the form of SNPs were predicted, and we identified 10,926,656 candidate genic SNPs, with 1,082,854 found in variable genes and 9,843,802 in core genes. A greater proportion of SNPs in variable genes were predicted to have an impact on protein structure and function, suggesting reduced selective pressure for these genes (Supplemental Figure S5). There was relatively wide genetic differentiation between *Ensete* and *Musa* genera as apparent from average Weir‐Cockerham's *F*_ST_ value of 0.29. Selective sweep analysis further identified regions in the pangenome that may explain the genetic differentiation among the two genera. Some genes in the highly differentiated regions have GO terms related to flavonoid biosynthesis, response to stress including defence response, meristem initiation, cell division, protein transport and refolding, as well as metabolite transport (Figure 4). The genes with the highest *F*_ST_ values are listed in Supplemental Table S10. The selective sweep analysis identified regions that may play roles in evolution and selection in the two genera and includes genes related to drought tolerance and auxin biosynthesis. *Ensete* plants are known to be tolerant to drought stress (Borrell et al., 2019), and understanding the genomic basis for this important trait may support the breeding of more drought‐tolerant *Musa* banana species.

Disease is a constant threat to banana cultivation, and the identification of resistance is a major target for breeding new varieties. A total of 965 RGAs were identified, including 702 receptor‐like kinase genes, 136 nucleotide binding‐site leucine rich repeat (NLR) genes, and 127 receptor‐like proteins (Supplemental Table S11). Of the 965 RGAs, the majority are found in the reference assembly and only 70 (5.7%) are located in the newly assembled contigs. These numbers are much lower than other pangenomes (Hurgobin et al., 2018; Zhao et al., 2018; Bayer et al., 2019) suggesting that there is very low diversity in disease resistance genes across *Musa* and *Ensete*. In total, 717 Musaceae RGAs were core genes (74%) and the remaining 248 were variable (26%). A dendrogram based on RGA PAVs results in distinct *Musa* and *Ensete* groups (Supplemental Figure S6). The presence of these genus‐specific RGAs can be explained by their evolutionary distance and differential pathogen pressure during their evolution. Wu et al. (2016) identified 117, 93, and 62 NLRs in *M. acuminata*, *M. balbisiana*, and *M. itinerans*, respectively, and the authors suggest the decreasing number of NLRs among these three species happened as a result of their geographical distribution, where during the transition from humid tropical to cool subtropical habitats, some NLRs may have become less abundant as a result of reduced selection pressure from tropical pathogens.

Recent studies on banana resistance to bacterial pathogens indicate variable responses and uncover some potential new sources of resistance among the studied plants. For example, three landraces of *Ensete* among 20 studied were found to be resistant to *Xanthomonas vasicola* pv. *musacearum* (Muzemil et al., 2019). A similar study from 72 diverse *Musa* cultivars identified several resistant genotypes, mostly with BB or hybrid AB genome types (Nakato et al., 2019). The B genome is known to be a source of disease resistance in banana breeding programs (Ssekiwoko et al., 2006; Tripathi et al., 2008), though some A genome *Musa* genotypes also showed tolerant reactions to *Xanthomonas vasicola* (Nakato et al., 2019).

In summary, the banana pangenome provides a cross‐genus census of the conserved and disposable gene content for banana. We found a core gene content of 18,288 genes across *Musa* and *Ensete* species, while the core gene content for *Musa* alone is 27,858 indicating the extent of the two genomes’ divergence. Variable genes were enriched for annotations related to flowering and meristem regulation and we identified candidate regions for drought resistance, meristem initiation, and stress resistance. This information provides the foundation for broader diversity and evolutionary studies and is a resource for the application of genomics for the improvement of these important crops.

## CONFLICT OF INTEREST

The authors declare no conflict of interest.

## Supporting information

Supplemental Figure S1: Ortholog genes content assessment using BUSCO for four available banana genome assemblies.
Supplemental Figure S2. PCA of the banana pangenome samples based on PAVs showing grouping based on two principal component axis that represent 72.9% of the sample variations.
Supplemental Figure S3. PCA analysis of SNP variation among the Musaceae family.
Supplemental Figure S4: Cluster dendrogram based on present‐absent variation. The two genera of banana are clearly separated.
Supplemental Figure S5: SNP distribution among variable and core gene regions.
Supplemental Figure S6. Cluster dendrogram based on the PAVs among resistance‐gene analogs showed a clear genus‐based grouping (*Ensete* group on the left, and *Musa* group on the right) and some differentiation among the *Musa* plants.

Supplemental Table S1: Short read data for Banana family.
Supplemental Table S2: Banana pan‐genome component statistics.
Supplemental Table S3. RNAseq data used for the gene annotation of the banana pangenome.
Supplemental Table S4: General statistics of assembled *Musa* genomes.
Supplemental Table S5: Summary statistics of BUSCO analysis for orthologous gene contents of four banana genome assemblies.
Supplemental Table S6: Comparison of BUSCO analysis between reference and pangenome.
Supplemental Table S7. Number of genes as identified by presence absence variations.
Supplemental Table S8. GO terms enriched in the variable genes among samples within *Musa* genus.
Supplemental Table S9. GO terms enriched in the variable genes among samples within Musaceae family.
Supplemental Table S10. List of genes with extreme FST values between *Musa* and *Ensete* subsets and their annotations.
Supplemental Table S11: Resistance Gene Analogs (RGAs) predicted in the banana pangenome.
