# Supplementary material for: The pangenome of banana highlights differences between genera and genomes
Source: Plant Genome. 2021 Jul 5;15(1):e20100. doi: 10.1002/tpg2.20100 (PMC12806902; doi:10.1002/tpg2.20100)
Supplement: Supplementary file 1 [file TPG2-15-e20100-s001.docx]

**Supplementary Table 1: Short read data for Banana family.**

| **No.** | **Sample Name** | **Species** | **Data Volume (Mbp)** | **Raw Read Coverage*** | **Data Source** | **SRA Sample** | **BioProject** | **PAV analysis** |
| --- | --- | --- | --- | --- | --- | --- | --- | --- |
| 1 | Ensete Jungle Seed | *Ensete ventricosum* | 38,529 | 85.6 | NCBI | SRS373715 | PRJNA170519 | yes |
| 2 | Bedadit | *Ensete ventricosum* | 16,795 | 37.3 | NCBI | SRS660424 | PRJNA252658 | yes |
| 3 | Derea | *Ensete ventricosum* | 25,780 | 57.3 | NCBI | SRS1719918 | PRJNA341828 | yes |
| 4 | Bile | *Musa x paradisiaca* | 42,025 | 93.4 | IAARD |  |  | yes |
| 5 | Calcutta-4 | *Musa acuminata* | 40,512 | 90.0 | IAARD |  |  | yes |
| 6 | Kole | *Musa acuminata* | 42,717 | 94.9 | IAARD |  |  | yes |
| 7 | Mas Kirana | *Musa acuminata* | 49,179 | 109.3 | IAARD |  |  | yes |
| 8 | Tanduk | *Musa x paradisiaca* | 33,489 | 74.4 | IAARD |  |  | yes |
| 9 | Lidi | *Musa acuminata* | 13,099 | 29.1 | IAARD |  |  | yes |
| 10 | BSK-30 | *Musa acuminata* | 14,378 | 32.0 | IAARD |  |  | yes |
| 11 | FHIA 25 | *Musa x paradisiaca* | 7,669 | 17.0 | IAARD |  |  | yes |
| 12 | DH-Pahang | *Musa acuminata* | 49,629 | 110.3 | NCBI | ERS187915 | PRJEB3375 | yes |
| 13 | PKW | *Musa balbisiana* | 27,121 | 60.3 | NCBI | SRS473718 | PRJNA216985 | yes |
| 14 | Tongkat Langit Maluku | *Musa Fe’i* | 22,301 | 49.6 | IAARD |  |  | yes |
| 15 | Yunan | *Musa itinerans* | 79,642 | 177.0 | NCBI | SRS1303720 | PRJNA312694 | yes |
| 16 | Mazia | *Ensete ventricosum* | 4,582 | 10.2 | NCBI | SRS1719042 | PRJNA344540 | no |
| 17 | Nechuwe | *Ensete ventricosum* | 5,358 | 11.9 | NCBI | SRS1719044 | PRJNA344540 | no |
| 18 | Nobo | *Ensete ventricosum* | 4,560 | 10.1 | NCBI | SRS1719045 | PRJNA344540 | no |
| 19 | Yako | *Ensete ventricosum* | 4,642 | 10.3 | NCBI | SRS1719047 | PRJNA344540 | no |
| 20 | Yanbule | *Ensete ventricosum* | 5,130 | 11.4 | NCBI | SRS1719048 | PRJNA344540 | no |
| 21 | Buffero | *Ensete ventricosum* | 4,744 | 10.5 | NCBI | SRS1719051 | PRJNA344540 | no |
| 22 | Onjamo | *Ensete ventricosum* | 6,266 | 13.9 | NCBI | SRS1719917 | PRJNA342253 | no |
| 23 | Manis Kerinci | *Musa acuminata* | 6,019 | 13.4 | IAARD |  |  | no |
| 24 | Muli | *Musa acuminata* | 4,482 | 10.0 | IAARD |  |  | no |
| 25 | Jaran | *Musa acuminata* | 2,867 | 6.4 | IAARD |  |  | no |

^a^ Based on 450 Mb size of the *M. acuminata* reference genome.

**Supplementary Table 2: Banana pan-genome component statistics.**

| **Iteration** | **Sample** | **Species** | **Genome type** | **Scaffold > 1Kb** | **N50** | **Scaffold length** | **Cumulative length** |
| --- | --- | --- | --- | --- | --- | --- | --- |
| 0 | DH Pahang (reference) | *Musa acuminata* | AA | 25 | 37.6 MB | 451,018,445 | 451,018,445 |
| 1 | Bile | *Musa x paradisiaca* | AB | 52,980 | 2.4 KB | 173,182,778 | 624,201,223 |
| 2 | Calcutta-4 | *Musa acuminata* | AA | 1,064 | 991 bp | 3,623,137 | 627,824,360 |
| 3 | Mas Kirana | *Musa acuminata* | AA | 1,249 | 1.4 KB | 3,355,090 | 631,179,450 |
| 4 | Pisang Tanduk | *Musa x paradisiaca* | AAB | 237 | 741 bp | 1,180,436 | 632,359,886 |
| 5 | BSK-30 | *Musa acuminata* | AA | 88 | 930 bp | 313,506 | 632,673,392 |
| 6 | Other *Musa* (Lidi, FHIA 25, Muli, Manis Kerinci, Jaran)* | *-* | - | 54 | 793 bp | 190,491 | 632,863,883 |
| 7 | Tongkat Langit Maluku | *Musa textilis (Fe’i)* | TT | 23,285 | 1.4 KB | 78,360,448 | 711,224,331 |
| 8 | Kole | *Musa acuminata* | AA | 333 | 677 bp | 1,865,095 | 713,089,426 |
| 9 | PKW | *Musa balbisiana* | BB | 1,268 | 3.3 KB | 5,283,814 | 718,373,240 |
| 10 | Yunnan | *Musa itinerans* | - | 28,007 | 1.2 KB | 96,308,220 | 814,681,460 |
| 11 | Ensete01 | *Ensete ventricosum* | - | 82,587 | 2.8 KB | 281,328,010 | 1,096,009,470 |
| 12 | Ensete Bedadit | *Ensete ventricosum* | - | 2,702 | 2.7 KB | 10,032,166 | 1,106,041,636 |
| 13 | Ensete Derea01 | *Ensete ventricosum* | - | 175 | 680 bp | 1,845,276 | 1,107,886,912 |
| 14 | Other *Ensete* (Buffero, Onjamo, Yako, Mazia Yanbule, Nobo, nechuwe)* | *Ensete ventricosum* | - | 3,206 | 860 bp | 15,441,851 | 1,123,328,763 |
|  | TOTAL |  |  | 197,260 |  | 1,123,328,763 |  |

* Sample reads were combined in one pangenome assembly iteration

**Supplementary Table 3. RNAseq data used for the gene annotation of the banana pangenome.**

| BioProject | Organism | Sample | Reads volume (MBases) | Count of runs |
| --- | --- | --- | --- | --- |
| PRJNA169175 | *Musa* ABB Group | Plantain | 7,910 | 1 |
| PRJNA208773 | *Musa acuminata* AAA Group | Brazilian | 4,389 | 1 |
| PRJNA210240 | *Musa acuminata* | Cavendish | 2,288 | 1 |
| PRJNA225660 | *Musa acuminata* AAA Group | MacuAAA | 8,827 | 1 |
| PRJNA242708 | *Musa balbisiana* | BeeHeeKela | 5,222 | 1 |
| PRJNA242709 | *Musa acuminata* | Calcutta4 | 4,872 | 1 |
| PRJNA287860 | *Musa acuminata* | Berangan | 50,601 | 9 |
| PRJNA311491 | *Musa* | Karthobiumtham | 13,965 | 2 |
|  |  | Nendran | 13,214 | 2 |
| PRJNA319058 | *Musa acuminata* AAA Group | Williams | 117,887 | 27 |
| PRJNA326055 | *Musa balbisiana* | PKW | 3,484 | 1 |
|  | *Musa beccarii* | BB2 | 8,083 | 1 |
| PRJNA327009 | *Musa acuminata* | AS1 | 3,825 | 1 |
|  |  | AS2 | 3,881 | 1 |
|  |  | WT | 4,784 | 1 |
| PRJNA81189 | *Musa acuminata* AAA Group | B1 | 4,415 | 1 |
|  |  | NK1 | 4,458 | 1 |
| Total |  |  | **262,105** | **53** |

**Supplementary Table 4: General statistics of assembled *Musa* genomes.**

| **Species** | **Variety** | **Assembly level** | **Assembly strategy** | **Assembly size** | **No. Scaffold ^2^** | **Scaffold N50** | **Reference** |
| --- | --- | --- | --- | --- | --- | --- | --- |
| *Musa acuminata* ssp. *malaccensis* | Doubled-haploid (DH), Pahang | Pseudo-chromosome | *De novo* | 472.2 Mb | 7,512 | 1,311,088 | D’Hont et al., 2012 |
| *Musa balbisiana* | Pisang Kluthuk Wulung (PKW) | Pseudo-chromosome ^1^ | Consensus mapping to *M. acuminata* assembly | 402.5 Mb |  |  | Davey et al., 2013 |
|  | Pisang Kluthuk Wulung (PKW) | Scaffold |  | 286 Mb | 53,523 | 10,338 |  |
| *Musa itinerans* | Yunnan | Scaffold | *De novo* | 462.1 Mb | 7,194 | 192,092 | Wu et al., 2016 |
|  | Yunnan | Pseudo-chromosome | Synteny mapping to *M. Acuminata assembly* | 273.2 Mb | 1,043 |  |  |
| *Musa schizocarpa* | ITC0926 | Pseudo-chromosome | *De novo*  *Long reads* | 525 Mb | 227 | 36,762,082 | Belser et al., 2018 |
| *Musa acuminata ssp. banksii* | Banksii | Scaffold | *De novo*  *Long reads* | 464 Mb | 9,467 | 435,833 |  |
| *Musa acuminata ssp. zebrina* | Maia Oa | Scaffold | *De novo* | 639 Mb | 56,481 | 37,689 |  |
| *Musa acuminata ssp. burmannica* | Calcutta 4 | Scaffold | *De novo* | 526 Mb | 47,753 | 22,183 |  |
| *Ensete ventricosum* | Bedadeti | Scaffold | *De novo* | 452 Mb | 45,742 | 21,097 | Harrison et al., 2014 |
| *Ensete ventricosum* | Onjamo | Scaffold | *De novo* | 445 Mb | 51,525 | 16,208 |  |
| *Ensete ventricosum* | Derea | Contig | *De novo* | 429 Mb | 60,129 | 12,314 |  |
| *Ensete ventricosum* | Jungle Seeds | Scaffold | *De novo* | 437 Mb | 52,691 | 13,866 |  |

^1^ scaffolded using *M. acuminata*

^2^ based on scaffold size >= 1kb^,^ except for *M. schizocarpa* which is based on scaffold size >= 2kb

**Supplementary Table 5: Summary statistics of BUSCO analysis for orthologous gene contents of four banana genome assemblies.**

| **BUSCO Category** | | ***Musa acuminata*** | ***Musa balbisiana*** | ***Musa itinerans*** | ***Musa schizocarpa*** |
| --- | --- | --- | --- | --- | --- |
| Complete (% total) | | 1329 (92.3%) | 1318 (91.5%) | 1318 (91.5%) | 1329 (92.3%) |
|  | Complete and single-copy | 1218 | 1209 | 1192 | 1174 |
|  | Complete and duplicated | 111 | 109 | 126 | 155 |
| Fragmented | | 35 | 35 | 36 | 27 |
| Missing | | 76 | 87 | 86 | 84 |
| Total | | 1440 | 1440 | 1440 | 1440 |

**Supplementary Table 6: Comparison of BUSCO analysis between reference and pangenome.**

| **Category** | ***M. acuminata* reference** | **Banana pangenome** |
| --- | --- | --- |
| Complete BUSCOs (C) | 1390 | 1391 |
| Complete and single-copy BUSCOs (S) | 912 | 909 |
| Complete and duplicated BUSCOs (D) | 478 | 482 |
| Fragmented BUSCOs (F) | 17 | 16 |
| Missing BUSCOs (M) | 33 | 33 |
| Total BUSCO groups searched | 1440 | 1440 |

**Supplementary Table 7. Number of genes as identified by presence absence variations.**

| **Sample** | **Species** | **Number of genes present** |
| --- | --- | --- |
| Bile | *Musa x paradisiaca* | 37091 |
| BSK30 | *M. acuminata* | 34638 |
| Calcutta | *M. acuminata* | 35298 |
| Ensete01 | *E. ventricosum* | 27452 |
| Ensete Bedadit | *E. ventricosum* | 29928 |
| Ensete Derea | *E. ventricosum* | 30817 |
| FHIA-25 | *M. acuminata* | 35460 |
| Itinerans | *M. itinerans* | 34877 |
| Kole | *M. acuminata* | 35328 |
| Lidi | *M. acuminata* | 34924 |
| Mas Kirana | *M. acuminata* | 35234 |
| Pahang | *M. acuminata* | 34999 |
| PKW | *M. balbisiana* | 34612 |
| Tanduk | *Musa x paradisiaca* | 36713 |
| Tongkat Langit Maluku | *M. textilis* | 32846 |
| ***Average*** |  | ***34014*** |

**Supplementary Table 8. GO terms enriched in the variable genes among samples within *Musa* genus.**

| Molecular Function* | | Biological Process* | |
| --- | --- | --- | --- |
| GO:0043531 | **ADP binding** | GO:0015074 | **DNA integration** |
| GO:0010333 | **terpene synthase activity** | GO:0002084 | protein depalmitoylation |
| GO:0008171 | **O-methyltransferase activity** | GO:0048441 | petal development |
| GO:0045735 | nutrient reservoir activity | GO:0048833 | specification of floral organ number |
| GO:0008474 | palmitoyl-(protein) hydrolase activity | GO:0006369 | termination of RNA polymerase II transcription |
| GO:0052692 | **raffinose alpha-galactosidase activity** | GO:0033609 | oxalate metabolic process |
| GO:0004506 | squalene monooxygenase activity | GO:0010219 | regulation of vernalization response |
| GO:0030145 | manganese ion binding | GO:0010951 | negative regulation of endopeptidase activity |
| GO:0046564 | oxalate decarboxylase activity | GO:0005983 | starch catabolic process |
| GO:0004866 | endopeptidase inhibitor activity | GO:0005987 | sucrose catabolic process |
| GO:0004523 | **RNA-DNA hybrid ribonuclease activity** | GO:0048442 | sepal development |
| GO:0000287 | **magnesium ion binding** | GO:0009767 | photosynthetic electron transport chain |
| GO:0004556 | **alpha-amylase activity** | GO:0043988 | histone H3-S28 phosphorylation |
| GO:0035175 | histone kinase activity (H3-S10 specific) | GO:0043987 | histone H3-S10 phosphorylation |
| GO:0044022 | histone kinase activity (H3-S28 specific) | GO:0048506 | regulation of timing of meristematic phase transition |

* Terms in bold are also found enriched among samples within genus *Musa* only (Supplementary Table 8).

**Supplementary Table 9. GO terms enriched in the variable genes among samples within *Musaceae* family.**

| Molecular Function* | | Biological Process* | |
| --- | --- | --- | --- |
| GO:0043531 | **ADP binding** | GO:0015074 | **DNA integration** |
| GO:0010333 | **terpene synthase activity** | GO:0000103 | sulfate assimilation |
| GO:0003735 | structural constituent of ribosome | GO:0006662 | glycerol ether metabolic process |
| GO:0008171 | **O-methyltransferase activity** | GO:0010466 | negative regulation of peptidase activity |
| GO:0004523 | **RNA-DNA hybrid ribonuclease activity** | GO:0006122 | mitochondrial electron transport, ubiquinol to cytochrome c |
| GO:0004556 | **alpha-amylase activity** | GO:0006414 | translational elongation |
| GO:0052692 | **raffinose alpha-galactosidase activity** | GO:0006767 | water-soluble vitamin metabolic process |
| GO:0030414 | peptidase inhibitor activity | GO:0042274 | ribosomal small subunit biogenesis |
| GO:0000287 | **magnesium ion binding** | GO:0006541 | glutamine metabolic process |
| GO:0070566 | adenylyltransferase activity | GO:0043631 | RNA polyadenylation |
|  |  | GO:0046352 | disaccharide catabolic process |

* Terms in bold are also found enriched among samples within genus *Musa* only (Supplementary Table 7).

**Supplementary Table 10. List of genes with extreme F_ST_ values between *Musa* and *Ensete* subsets and their annotations.**

| **Chromosome/contig** | **Gene** | **Annotation** | **GO** | **GO-function** | **More Info** |
| --- | --- | --- | --- | --- | --- |
| **F_ST_ with weighted values: -1** | | | | | |
| Bile_85317 | gene40131 |  |  |  |  |
| Itinerans_134698 | gene187877 |  |  |  |  |
| chr01 | gene218979 | Putative Anaphase-promoting complex subunit cdc20 | GO:0010997 | cell division cycle cofactor of APC complex-like | Component of the anaphase promoting complex/cyclosome (APC/C), a cell cycle-regulated E3 ubiquitin-protein ligase complex that controls progression through mitosis and the G1 phase of the cell cycle. |
| chr01 | gene218980 | Hypothetical protein |  |  |  |
| chr01 | gene218981 | Putative Exopolygalacturonase | GO:0016787 | exopolygalacturonase-like | Cell wall modification |
| chr04 | gene228961 | uncharacterized LOC103973729 |  |  |  |
| chr04 | gene228962 | uncharacterized LOC103973728 | GO:0005634 | RWP-RK domain containing |  |
| chr04 | gene228963 | uncharacterized protein yae1-like |  |  |  |
| **F_ST_ with weighted values: 1** | | | | | |
| Bile_106984 | gene8427 |  | GO:0016021 | transmembrane 87A-like |  |
| Bile_114439 | gene20494 |  | GO:0008270 | E3 ubiquitin- ligase RGLG2-like | E3 ubiquitin-protein ligase that mediates the formation of 'Lys-63'-linked ubiquitin chains. Regulates apical dominance by acting on the auxin transport proteins abundance (PubMed:17586653). Mediates ubiquitination and subsequent proteasomal degradation of ERF053 in response to drought stress. Acts as a negative regulator of drought stress response (PubMed:22095047). |
| Ensete01_75678 | gene128631 |  |  |  |  |
| TongkatLangitMaluku_69823 | gene214666 |  |  |  |  |
| TongkatLangitMaluku_69823 | gene214668 |  | GO:0009535 | photosystem II K (chloroplast) |  |

**Supplementary Table 11: Resistance Gene Analogs (RGAs) predicted in the banana pangenome.**

| **Type of RGA** | **Class** | **Count** | **Class-count** |
| --- | --- | --- | --- |
| CN | nucleotide binding-site leucine rich repeat (NLR) | 7 | 136 |
| CNL |  | 58 |  |
| NBS |  | 16 |  |
| NL |  | 49 |  |
| TN |  | 2 |  |
| TX |  | 4 |  |
| RLK-LRR | receptor-like kinase (RLK) | 323 | 702 |
| RLK-LysM |  | 8 |  |
| RLK-other receptor |  | 371 |  |
| RLP-LRR | receptor-like proteins (RLP) | 125 | 127 |
| RLP-LysM |  | 2 |  |
| Total |  |  | 965 |
