# Supplementary material for: The pangenome of banana highlights differences between genera and genomes
Source: Plant Genome. 2021 Jul 5;15(1):e20100. doi: 10.1002/tpg2.20100 (PMC12806902; doi:10.1002/tpg2.20100)
Supplement: Supplementary file 2 [file TPG2-15-e20100-s002.docx]

# **Supplementary Figures**

Supplementary Figure 1: Ortholog genes content assessment using BUSCO for four available banana genome assemblies.

Supplementary Figure 2. PCA of the banana pangenome samples based on PAVs showing grouping based on two principal component axis that represent 72.9% of the sample variations. PCA clustering of the samples within the *Musaceae* family indicates sub-groupings that reflect genus and species division (colored differently, Ev: *E. ventricosum*; Ma: *M. acuminata*; Mb: *M. balbisiana*; Mi: *M. itinerans*; Mt: *M. textilis*) as well as genome type traits (coded as point shapes).

Supplementary Figure 3. PCA analysis of SNP variation among the Musaceae family. Three main clusters are evident, one is specific to *Ensete* species (purple, Ev, *Ensete ventricosum*), another one is formed by sub-species of *Musa acuminata* (green, Ma, *Musa acuminata*) and the last one contains the rest of the species (Mt (*Musa textilis*), Mi (*Musa itinerans*), Mb (*Musa balbisinia*)).

Supplementary Figure 4: Cluster dendrogram based on present-absent variation. The two genera of banana are clearly separated. The *Musa* genus cluster is further divided into sub-clusters that generally reflect the species groupings or genome-types.

Supplementary Figure 5: SNP distribution among variable and core gene regions.

Supplementary Figure 6. Cluster dendrogram based on the PAVs among resistance-gene analogs showed a clear genus-based grouping (*Ensete* group on the left, and *Musa* group on the right) and some differentiation among the *Musa* plants.
